# Infectious Healthcare Waste Management Practices and Associated Factors Among Waste Handlers in Addis Ababa, Ethiopia: A Cross‐Sectional Study

**DOI:** 10.1002/hsr2.73036

**Published:** 2026-08-11

**Authors:** Trhas Tadesse Berhe, Agezegn Asegid Mekonen, Mekoya Mengistu Dabulo, Getachew Woldeyohonas Tedila

**Affiliations:** ^1^ School of Public Health, Public Health Department Yekatit 12 Hospital Medical College Addis Ababa Ethiopia; ^2^ School of Nursing Wachemo University College of Medicine and Health Sciences Hossana Ethiopia; ^3^ School of Medicine, Internal Medicine Department Yekatit 12 Hospital Medical College Addis Ababa Ethiopia

**Keywords:** Ethiopia, healthcare facilities, infectious healthcare waste, occupational safety, waste handlers

## Abstract

**Background and Aims:**

Infectious healthcare waste (IHCW) poses significant risks to healthcare workers, waste handlers, patients, and the environment. Despite growing awareness, suboptimal waste management practices remain common in Ethiopia, particularly among frontline waste handlers directly exposed to infectious materials. This study aimed to assess the magnitude of proper IHCW management practices among waste handlers in health facilities in Addis Ababa and to identify factors associated with these practices.

**Methods:**

A facility‐based cross‐sectional study was conducted from July 1 to August 30, 2023, among healthcare waste handlers in selected public and private health facilities in Addis Ababa, Ethiopia. Health facilities were selected using proportional allocation and simple random sampling. A total of 422 waste handlers were sampled from the selected facilities using proportional allocation followed by simple random sampling, of whom 379 participated in the study. Data were collected using a structured interviewer‐administered questionnaire, a direct observation checklist, and a review of relevant waste management documents. Descriptive statistics were used to summarize infectious healthcare waste management practices, and bivariable and multivariable binary logistic regression analyses were performed to identify factors associated with proper infectious healthcare waste management practices. Adjusted odds ratios (AORs) with 95% confidence intervals (CIs) were calculated, and statistical significance was declared at *p* < 0.05.

**Results:**

Overall, 218 of 379 waste handlers (57.5%; 95% CI: 52.4%–63.1%) demonstrated proper infectious healthcare waste management (IHCWM) practices. Most facilities had designated waste storage areas (349/379, 92.1%) and separate containers for hazardous and non‐hazardous waste (350/379, 92.3%). However, gaps were observed in waste labeling, the appropriate placement of waste containers, and the use of protective footwear among waste handlers. In multivariable logistic regression analysis, work experience of 3–5 years (AOR = 0.56; 95% CI: 0.33–0.97), awareness of healthcare waste management guidelines (AOR = 2.35; 95% CI: 1.21–4.57), availability of adequate personal protective equipment (PPE) (AOR = 0.59; 95% CI: 0.35–0.97), and reporting occupational injuries to management (AOR = 0.50; 95% CI: 0.28–0.92) were significantly associated with proper IHCWM practices.

**Conclusion:**

Although more than half of the waste handlers practiced proper IHCW management, critical gaps remain in training, guideline implementation, and PPE provision. Strengthening institutional policies, providing targeted training, and ensuring consistent availability of PPE are essential for improving occupational safety and promoting effective healthcare waste management in Addis Ababa.

## 1 Background

Infectious healthcare waste management (IHCWM) is a vital component of safe and effective healthcare delivery, encompassing all procedures related to the generation, segregation, collection, transportation, storage, treatment, and final disposal of waste produced in healthcare facilities [1]. Infectious healthcare waste includes potentially infectious materials such as sharps, contaminated dressings, body fluids, pathological waste, and laboratory materials. These wastes require careful handling due to their potential to transmit infectious agents within healthcare settings. Inadequate management of infectious healthcare waste remains a major concern in many health systems, particularly in low‐resource settings where segregation, handling, and disposal practices may be inconsistent. This increases the likelihood of occupational exposure among healthcare workers and waste handlers, as well as environmental contamination and public health risks [2]. From a One Health perspective, poor healthcare waste management may lead to soil contamination through unsafe dumping and landfill leakage, water contamination through the improper disposal of infectious fluids, and air pollution resulting from the open burning of healthcare waste. Such contamination can further affect agricultural systems by causing crops to take up hazardous substances and pose risks to animals, including scavenging livestock and stray animals exposed to infectious waste, thereby facilitating pathogen transmission across the human, animal, and environmental domains [1].

Worldwide, healthcare waste generation is increasing, with hospitals in high‐income countries producing substantially more waste per hospital bed per day than those in low‐ and middle‐income countries. According to the World Health Organization (WHO), hazardous healthcare waste generation averages up to 0.5 kg per bed per day in high‐income countries, compared with approximately 0.2 kg per bed per day in low‐income countries, although generation rates may vary depending on segregation practices and facility type [3].

However, some studies have reported that high‐income hospitals may generate as much as 8–10 kg of healthcare waste per bed per day [3]. In Africa, healthcare waste generation rates are generally lower, ranging from 0.4 to 2.5 kg per bed per day, with some studies reporting an average of approximately 0.8 kg per bed per day [3]. In Ethiopia, healthcare waste generation has been estimated at approximately 1.1 kg per bed per day [4]. Despite the relatively lower quantities of healthcare waste generated in low‐income settings, inadequate healthcare waste management, resulting from limited training, insufficient personal protective equipment, and inadequate disposal infrastructure, poses significant health and environmental risks [5, 6, 7].

In Ethiopia, including Addis Ababa, healthcare waste management remains suboptimal, particularly among waste handlers whose activities involve procedures that expose them directly to infectious materials [8]. Unsafe handling of infectious waste contributes to occupational hazards, including needle‐stick injuries and exposure to bloodborne pathogens such as hepatitis B, hepatitis C, and HIV [9, 10]. Studies indicate that a single needle‐stick injury can increase the risk of hepatitis B by 30%, hepatitis C by approximately 1.8%, and HIV by about 0.3% [11]. Waste handlers, including cleaners, porters, and other support staff in healthcare facilities, frequently work without adequate training or personal protective equipment, making them highly vulnerable to these infections [12]. While previous research has primarily focused on healthcare professionals, the knowledge, practices, and challenges faced by waste handlers remain largely underexplored, representing a critical evidence gap for policy and intervention planning [12].

Quantitative evidence on the practices of healthcare waste handlers is essential for designing targeted interventions to improve infectious healthcare waste management. Evaluating their practices, risks, and compliance with recommended procedures provides actionable information for policymakers, facility administrators, and public health authorities. In Addis Ababa, where the rapid expansion of healthcare facilities is accompanied by inadequate regulatory oversight and limited access to training, the risk of occupational hazards and environmental contamination is of particular concern. A systematic assessment of waste handlers' practices can guide the development of context‐specific interventions aimed at improving occupational safety, environmental protection, and the overall effectiveness of healthcare waste management in the city.

This study, therefore, aims to generate evidence on the current status of infectious healthcare waste handling practices among frontline waste handlers, identify gaps in knowledge, training, and adherence to recommended waste management protocols, and provide a foundation for the development of context‐specific interventions and training programs tailored to their needs. By doing so, it will support policymakers and healthcare administrators in developing and implementing strategies to improve occupational safety and reduce the risk of infection. Improving infectious healthcare waste management among waste handlers represents a public health priority, and this study addresses a critical evidence gap by providing a quantitative assessment of current practices in health facilities in Addis Ababa, thereby generating actionable evidence to inform interventions that protect healthcare workers, patients, communities, and the environment.

## 2 Methods

### 2.1 Study Area and Period

The study was conducted in Addis Ababa, the capital city of Ethiopia, from July 1 to August 30, 2023. Addis Ababa was purposively selected because it serves as the national referral and administrative hub, hosting a wide range of healthcare facilities with varying levels of service delivery, infrastructure, and waste management capacity. As the city with the highest concentration of healthcare services in the country, it generates a substantial volume and diversity of healthcare waste due to high patient flow and service intensity, making it a suitable setting for assessing infectious healthcare waste management practices.

The city has 12 public hospitals, 98 public health centers, 268 specialty clinics, 318 medium clinics, 152 primary clinics, and 738 private clinics, serving approximately 35,000 healthcare providers and 11,234 healthcare waste handlers (674 healthcare waste handlers were from private facilities) [13]. In addition, the coexistence of public and private health facilities with differing resource availability and waste handling systems allows for a comprehensive assessment of healthcare waste management practices across multiple service levels.

### 2.2 Study Design

A facility‐based cross‐sectional quantitative design was employed to assess infectious healthcare waste management practices among waste handlers.

### 2.3 Source Population

The source population consisted of all healthcare waste handlers working in public and private health facilities in Addis Ababa, Ethiopia, during the study period.

### 2.4 Study Population

The study population comprised healthcare waste handlers in Addis Ababa, Ethiopia, who were directly involved in waste management activities, including cleaners, porters, and operational staff responsible for handling healthcare waste within selected health facilities in the city, and who fulfilled the inclusion criteria.

### 2.5 Eligibility Criteria

#### 2.5.1 Inclusion Criteria

Health care waste handlers and managers working in selected public and private health facilities in Addis Ababa, Ethiopia, during the study period who were directly involved in healthcare waste management activities and who provided informed consent were included in the study.

#### 2.5.2 Exclusion Criteria

Healthcare workers who were not directly involved in healthcare waste handling activities, those who were on leave or absent during the data collection period, and individuals who declined to participate were excluded from the study.

### 2.6 Sample Size and Sampling Procedure

The sample size for health facilities was determined using the single population proportion formula:

$$n=\frac{\left({Z_{\alpha /2})}^{2}p(1-p\right)}{d^{2}}$$

where *n* is the required sample size, *Z* = 1.96 at the 95% confidence level, *p* = 0.21 (the proportion of health facilities with good healthcare waste management practices reported by Yazie et al. [14], and *d* = 0.05 (margin of error). The initial sample size was 255 health facilities. Since the total number of health facilities in Addis Ababa was 1586 (i.e., less than 10,000), the finite population correction (FPC) was applied using:

$$n_{f}=\frac{n}{1+\frac{n}{N}}$$

where *N* = 1586 is the total number of health facilities in Addis Ababa. After applying the finite population correction, the final sample size was 219 health facilities. The selected health facilities were proportionally allocated according to facility ownership (public and private) and facility type, and facilities within each stratum were selected using simple random sampling from the available facility list obtained from the Addis Ababa health administration.

**Figure 1:**
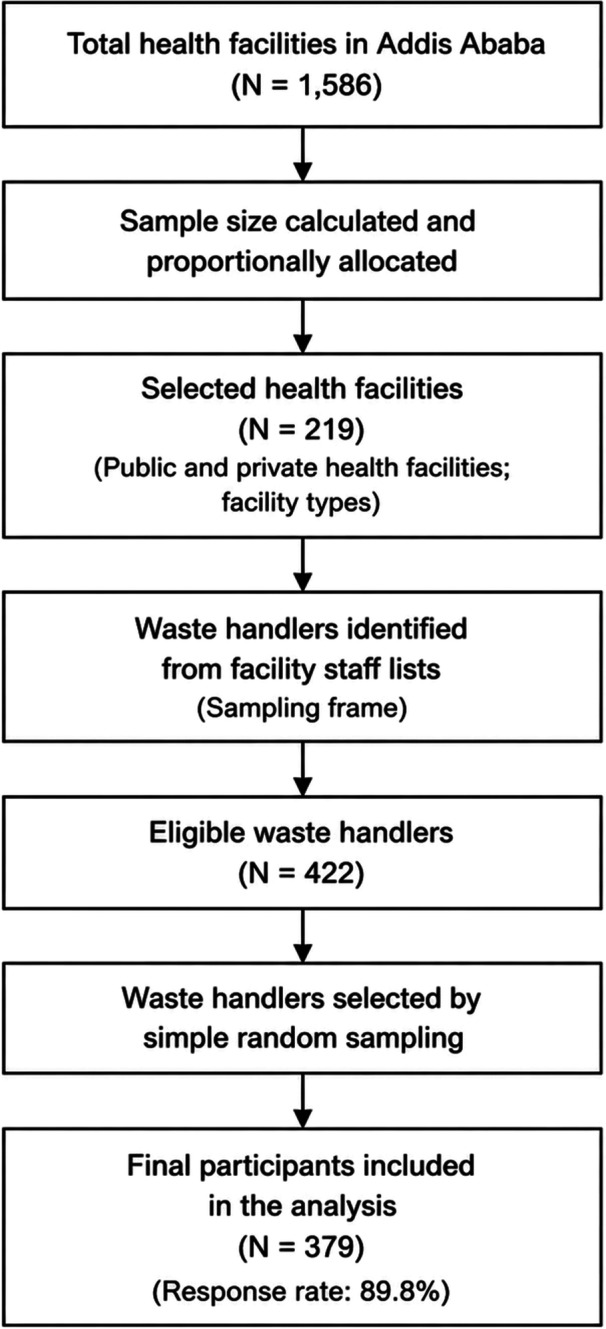
Flow diagram of health facility and participant selection for the study conducted among waste handlers in Addis Ababa, Ethiopia, 2023.

Within the selected health facilities, a total of 422 waste handlers were selected for the study. The sample size was determined using the single population proportion formula:

$$n=\frac{\left({Z_{\alpha /2})}^{2}p(1-p\right)}{d^{2}}$$

where *n* is the required sample size, *Z* = 1.96 at the 95% confidence level, *p* = 0.50 (assuming 50% prevalence of proper waste management practice due to the absence of previous studies), and *d *= 0.05 margin of error. A 10% non‐response rate was added to the calculated sample size, resulting in a final sample size of 422. Because the source population exceeded 10,000, the finite population correction was not applied. The lists of healthcare waste handlers were obtained from each participating health facility and served as the sampling frame. The sample was proportionally allocated according to facility type and ownership, and participants were selected using simple random sampling (Figure 1).

### 2.7 Study Variables

The dependent variable for this study was the infectious healthcare waste management practice. The practice was categorized as safe or unsafe based on the operational definition described below. Independent variables included socio‐demographic characteristics (age, sex, education, experience, profession) and context‐related factors (facility type, ownership, training, supervision, availability of equipment, and policies).

### 2.8 Data Collection Methods, Techniques, and Tools

Data were collected using a structured, interviewer‐administered questionnaire adapted from WHO guidelines and relevant literature, capturing knowledge, attitudes, and practices related to infectious waste handling [15, 16, 17]. The questionnaire was translated from English into the local working language (Amharic) and then back‐translated into English to ensure consistency and conceptual equivalence. Practice‐related information was assessed using eight infectious healthcare waste management practice items. Each correct practice response was assigned a score of 1, and an incorrect response a score of 0. The reported practice responses were verified through direct observation using a standardized checklist during the facility visit. The final practice score was generated based on the verified responses, where observed practices were used to confirm and recode the corresponding questionnaire responses.

Direct observations were conducted during the data collection period at the health facilities using a standardized checklist to verify self‐reported infectious healthcare waste management practices. The checklist assessed key practices, including waste segregation, use of personal protective equipment, availability and utilization of waste management equipment, supervision mechanisms, and adherence to institutional healthcare waste management policies and guidelines. Only practice responses confirmed through direct observation were considered for the final practice score and classification. Observers were trained on the standardized checklist and observation procedures; however, they were not blinded to the study objectives.

Secondary data, including waste transfer forms and incineration logs, were reviewed to validate waste management activities. The tool was pre‐tested among 17 waste handlers in health facilities with similar settings before the actual data collection, and feedback from waste handlers was incorporated to improve the clarity, wording, and cultural appropriateness of the questions. Data collectors were trained for 1 day on study procedures, ethical conduct, observation techniques, and electronic data capture. A supervisor conducted spot checks and daily reviews to ensure data completeness and consistency. The use of the Amharic version improved understanding among waste handlers with limited English proficiency, thereby improving data accuracy and reliability.

### 2.9 Data Analysis

Quantitative data were exported from the Computer‐Assisted Personal Interview (CAPI) system into SPSS version 23 (IBM Corp., Armonk, NY, USA) for analysis. Continuous variables were summarized using means ± standard deviation (SD) for normally distributed data and medians with interquartile ranges (IQR) for non‐normally distributed data. Categorical variables were summarized using frequencies and percentages.

Descriptive statistics, including frequencies, percentages, and cross‐tabulations, were used to summarize infectious healthcare waste handling practices and facility characteristics. Bivariable binary logistic regression analysis was performed to assess the association between each independent variable and safe infectious healthcare waste handling practice. Variables with a *p* value ≤ 0.05 in the bivariable analysis, together with variables identified a priori based on previous literature and their relevance to the study objectives, were entered into the multivariable binary logistic regression model using the enter method. A total of 13 independent variables were included in the final multivariable model. Adjusted odds ratios (AORs) with 95% confidence intervals (CIs) were reported, and statistical significance was declared at *p* < 0.05.

Before fitting the multivariable model, multicollinearity among independent variables was assessed using tolerance and the variance inflation factor (VIF), and no evidence of problematic multicollinearity was observed. The number of outcome events was considered during model development; 218 participants had safe infectious healthcare waste handling practices, providing approximately 17 events per variable for the 13 predictors included in the final model, indicating an adequate number of outcome events relative to the number of variables. Model fit was assessed using the Hosmer–Lemeshow goodness‐of‐fit test, with *p* ≥ 0.05 indicating adequate model fit (*χ* ^2 ^= 8.21, *p* = 0.41). All analyses were prespecified based on the study objectives, and no exploratory subgroup analyses were conducted.

### 2.10 Operational Definitions

Infectious waste management practice: the systematic approach to handling, treating, and disposing of waste that poses an infection risk, including segregation, collection, storage, treatment, and final disposal [4, 16, 18].

Practice of infectious healthcare waste (IHCW) management: In this study, the practice of infectious healthcare waste management among healthcare waste handlers was assessed using eight practice‐related items and dichotomized as either “safe” or “unsafe.” Each correct response was scored as 1, and each incorrect response as 0. Respondents who scored six or more out of eight items (≥ 75%), verified through observation, were classified as having safe practice, while those who scored less than six (< 75%) were classified as having unsafe practice [19]. This cut‐off was chosen to reflect a high level of compliance with recommended waste management practices.

The practice items included daily emptying of infectious healthcare waste, proper closing and handling of sharps containers, correct segregation of waste by type, appropriate recycling practices, use of heavy‐duty gloves, wearing protective footwear, washing gloves after use, and adherence to proper waste disposal procedures [15].

Knowledge of infectious healthcare waste (IHCW) management: Health professionals' knowledge of infectious healthcare waste management was assessed using 17 questions covering key areas, including separation, collection, storage, and disposal practices. Respondents were considered knowledgeable if they correctly answered 12 or more questions, and not knowledgeable if they answered fewer than 12. Each correct response was scored as 1, and incorrect responses as 0. This assessment allowed for a standardized evaluation of the knowledge levels of healthcare professionals regarding safe and effective management of infectious healthcare waste [4, 18].

### 2.11 Data Quality Assurance

Multiple measures were implemented to ensure the quality, validity, and reliability of the data. The data collection tools (questionnaire and observational checklists) were adapted from WHO guidelines and relevant literature to ensure content validity. The instruments were reviewed by experts in public health and environmental health to ensure relevance, clarity, and completeness.

The questionnaire was translated from English into Amharic and then back‐translated into English to ensure conceptual equivalence and consistency. A pre‐test was conducted in health facilities with similar characteristics to the study setting to assess clarity, flow, cultural appropriateness, and feasibility. Feedback from waste handlers during the pre‐test was incorporated to refine the tools.

Internal consistency of the instruments was assessed using Cronbach's alpha, which showed acceptable reliability for both practice (*α* = 0.87) and knowledge (*α* = 0.78) sections. Since the knowledge and practice items were designed to measure single constructs based on established WHO guidelines, factor analysis was not performed.

Data collectors and supervisors received intensive training on study objectives, ethical considerations, and standardized data collection procedures. Daily supervision, spot‐checking, and review of completed questionnaires were conducted to ensure completeness and consistency. In addition, triangulation with secondary records (waste transfer forms and incineration logs) was used to improve data accuracy. The use of electronic data capture (CAPI) further minimized data entry errors and improved data quality.

### 2.12 Ethical Considerations

This study is part of the “Evaluation of Infectious Waste Management Among Health Facilities of Addis Ababa for the Development of an Interventional Framework,” conducted in Addis Ababa, Ethiopia, in 2023. Ethical approval was obtained from the Institutional Review Board (IRB) of Yekatit‐12 Hospital Medical College and the Addis Ababa Health Bureau Public Health Research and Emergency Management Directorate (Ref. No: Y12‐337/300/42). Permission to conduct the study was also granted by the Addis Ababa Health Bureau.

Informed consent was obtained from all participants before data collection. Confidentiality and anonymity of participants were strictly maintained throughout the study. The study adhered to ethical principles of voluntary participation, non‐discrimination, and protection of participants' rights. Data collectors and supervisors ensured culturally appropriate and gender‐sensitive data collection while minimizing any potential risks to participants.

## 3 Result

### 3.1 Characteristics of the Waste Handlers

A total of 422 waste handlers were initially selected, of whom 379 responded, giving a response rate of (379/420, 89.8.2%). The study population was predominantly female (98.2%). The largest age group was participants aged ≥ 35 years, while the smallest proportions were aged ≤ 24 years. More than half of the respondents had primary‐level education (197/379, 52.0%), whereas the smallest proportion had vocational education or above (59/379, 15.5%). Regarding work experience, the highest proportion of participants had ≥ 6 years of experience (140/379, 37.0%), while the lowest had ≤ 2 years (107/379, 28.2%). Nearly half of the respondents were working in general hospitals and health centers, and the vast majority were employed in public health facilities (356/379, 93.9%).

The mean age of respondents was 30.96 ± 8.24 years, with a median of 29 years (IQR: 25–35 years). The mean work experience in health facilities was 4.90 ± 3.46 years, and the mean duration of engagement in healthcare waste management was 4.61 ± 3.40 years (Table 1).

**Table 1:** Socio‐demographic and work‐related characteristics of waste handlers in public and private health facilities in Addis Ababa, Ethiopia, 2023 (*n* = 379).

Variables		Frequency	Percent
Sex	Female	372	98.2
	Male	7	1.8
Age in years	≤ 24	84	22.2
	25–29	116	30.6
	30–4	61	16.1
	≥ 35	118	31.1
Educational status	Primary (grade 1–8)	197	52
	Secondary (grade 9–12)	123	32.5
	Vocational and above	59	15.5
Current working department	OPDs	115	30.3
	Wards	123	32.5
	Laundry and morgue service	95	25.1
	Emergency	46	12.1
Work experience in years	≤ 2	107	28.2
	3–5	132	34.8
	≥ 6	140	37.0
Duration (in years) of engagement in waste handling process	≤ 2	123	32.5
	3–5	120	31.7
	≥ 6	136	35.8
Types of health facilities	General hospital	180	47.5
	Health center	185	48.8
	Others (clinic, and dermatology specialty center)	14	3.7
Ownership of the health facilities	Public	356	93.9
	Private	23	6.1

*Note:* Data are presented as frequency (*n*) and percentage (%). Percentages are based on the total sample size (*n* = 379).

Abbreviation: OPD, outpatient department.

### 3.2 Knowledge of Healthcare Waste Segregation, Collection and Storage

Knowledge of waste segregation and handling varied across domains. A high proportion correctly identified disposal methods for non‐infectious waste (305/379, 80.5%) and sharps waste (274/379, 72.3%). However, knowledge was limited for chemical waste (64/379, 16.9%) and radioactive waste (125/379, 33.0%).

Most respondents reported appropriate collection practices, including collecting sharps containers when three‐quarters full (302/379, 79.7%) and daily collection of infectious waste (258/379, 68.1%).

Storage practices were generally appropriate, with 297/379 (78.4%) reporting storage of infectious waste for no more than 1 day (Table 2).

Overall, 115 of 379 healthcare workers (30.3%; 95% CI: 26.1%–35.1%) had adequate knowledge of infectious waste management, based on the 17 selected knowledge‐related questions.

**Table 2:** Knowledge of healthcare waste segregation, collection, storage, and disposal practices among waste handlers in health facilities in Addis Ababa, Ethiopia, 2023 (*n* = 379).

Variable	Frequency	Percentage
Knowledge of infectious healthcare waste segregation		
Non‐infectious waste in black bags/bins	305	80.5%
Highly infectious waste in red biohazard containers	201	53.0%
Chemical waste in brown bags/bins	64	16.9%
Radioactive waste in yellow radioactive containers	125	33.0%
Sharp waste in yellow‐marked sharp biohazard containers	274	72.3%
High metal‐containing waste in red secured containers	51	13.5%
Non‐chemical liquid waste in red flask containers	63	16.6%
Knowledge of collection times for different types of healthcare waste		
Sharps waste collected when 3/4 full	302	79.7%
Infectious waste collected daily	258	68.1%
General waste collected daily	266	70.2%
Food waste collected daily	284	74.9%
Anatomical waste collected immediately	240	63.3%
Knowledge on healthcare waste storage time		
Sharps waste stored for up to 1 day	170	44.9%
Infectious waste stored for up to 1 day	297	78.4%
General waste stored for up to 1 day	274	72.3%
Food waste stored for up to 1 day	303	79.9%
Anatomical waste stored immediately	227	59.9%
Knowledge on disposal methods		
Sharp waste disposal methods: incineration only	245	64.6%
Infectious waste disposal method: incineration only	205	54.1%
General waste disposal methods: incineration only	188	49.6%
Food waste disposal methods: off‐site disposal, compost, or burial	220	58.0%
Improper color‐coding increases infectious waste	370	97.6%
Improper color‐coding contributes to improper disposal	369	97.4%
Mixing infectious with non‐infectious waste contributes to disease transmission	375	98.9%
Improper HCW disposal contributes to disease transmission	371	97.9%
Disposing of HCW without treatment contributes to disease transmission	366	96.6%

*Note:* Data are presented as frequency (*n*) and percentage (%), based on the total respondents (*n* = 379). Higher percentages indicate better knowledge or compliance with recommended healthcare waste management practices.

### 3.3 Infectious Waste Handling Practices Among Waste Handlers

At the facility level, most respondents reported the availability of separate waste storage areas (349/379, 92.1%) and separate containers for hazardous and non‐hazardous waste (350/379, 92.3%). However, 84/379 (22.2%) reported that not all waste containers were clearly labeled, and 75/379 (19.8%) indicated that containers were not always located in appropriate areas.

Use of personal protective equipment varied: 324/379 (84.5%) of waste handlers consistently used heavy‐duty gloves, while only 190/379 (50.1%) reported consistent use of protective footwear. Additionally, 305/379 (80.5%) reported washing gloves after use, and 350/379 (92.3%) reported washing hands after handling healthcare waste (Table 3).

**Table 3:** Infectious healthcare waste handling practices among waste handlers in health facilities in Addis Ababa, Ethiopia, 2023 (*n* = 379).

Variable	Frequency	Percentage
Have a separate healthcare waste storage area	349	92.1%
Have a separate container for hazardous and non‐hazardous waste	350	92.3%
All waste containers are clearly marked or labeled	295	77.8%
All waste containers located in the appropriate area	304	80.2%
Container leak‐proof materials for waste disposal	361	95.3%
Waste containers are easy to carry	350	92.3%
Sharp container puncture‐resistant material	354	93.4%
Healthcare waste containers are emptied daily or when 3/4 full	352	92.9%
Formal or informal waste separation	319	84.1%
Used plastic and intravenous sets are kept separately for recycling	233	61.5%
Wear heavy‐duty gloves all the time when handling medical waste	324	84.5%
Wear sturdy shoes when handling medical waste	190	50.1%
Wash your heavy‐duty gloves after handling HCW	305	80.5%
Wash your hands after handling HCW	350	92.3%

*Note:* Data are presented as frequency (*n*) and percentage (%), based on the total sample (*n* = 379). Percentages reflect the proportion of respondents reporting each practice.

**Figure 2:**
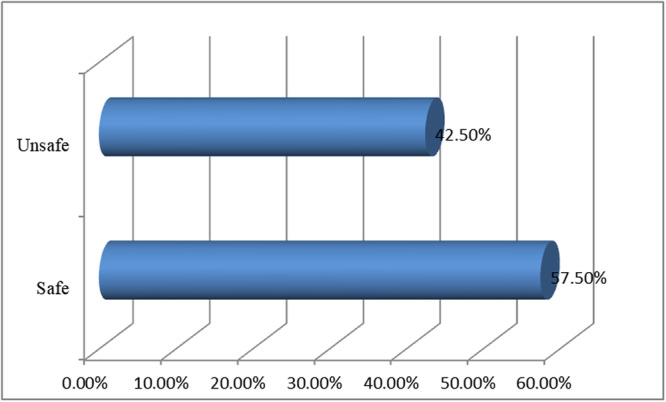
Proportion of waste handlers with safe and unsafe infectious healthcare waste handling practices in Addis Ababa, Ethiopia, 2023 (*n* = 379).

Overall, 218/379 waste handlers (57.5%; 95% CI: 52.4%–63.1%) demonstrated safe infectious healthcare waste management practices, indicating that a substantial proportion did not meet recommended safety standards (Figure 2).

### 3.4 Knowledge and Awareness of Healthcare Waste Handlers

Among respondents, 197/379 (52.0%) reported receiving training on healthcare waste management within the last 12 months. More than half (206/379, 54.4%) reported the availability of waste management rules and regulations, while 68/379 (17.9%) indicated that such rules were not practiced in their facilities. Similarly, 198/379 (52.2%) reported the presence of clearly defined procedures for waste handling, whereas 52/379 (13.7%) reported their absence, and 129/379 (34.0%) were unsure. Approximately half (192/379, 50.7%) reported that training was provided to newly hired staff. The majority (287/379, 75.7%) reported the presence of a waste management or infection prevention and control (IPC) committee, and 279/379 (73.6%) indicated that these committees were functional. Awareness of healthcare waste management guidelines was reported by 166/379 (43.8%) of participants. Only 139/379 (36.7%) reported adequate availability of personal protective equipment, while 240/379 (63.3%) reported an inadequate supply. In the previous 12 months, 68/379 (17.9%) reported experiencing waste‐related injuries. Almost all participants (369/379, 97.4%) agreed that improper color‐coding increases injury risk, and 371/379 (97.9%) reported that their facilities practiced waste segregation (Table 4).

**Table 4:** Knowledge and awareness of healthcare waste management among waste handlers in Addis Ababa health facilities, Ethiopia, 2023 (*n* = 379).

Variable	Frequency	Percentage
Training on infectious waste management (last 12 months)	197	52.0%
Availability of rules and regulations in the facility	206	54.4%
Defined procedures for waste handling	198	52.2%
Training for newly hired staff	192	50.7%
Waste management/IPC team in the facility	287	75.7%
Functional infectious waste management team	289	76.3%
Aware of infectious waste management guidelines	166	43.8%
Adequate personal protective equipment	139	36.7%
Experienced waste handling injury (last 12 months)	68	17.9%
Reported injury to management	80	21.1%
Improper color coding increases injury risk	369	97.4%
Facility segregates waste	371	97.9%
Regular infectious waste management training for all staff	169	44.6%

*Note:* Data are presented as frequency (*n*) and percentage (%), based on the total sample (*n* = 379).

### 3.5 Factors Associated With Infectious Waste Management Practice Among Waste Handlers

In multivariable logistic regression analysis, work experience, awareness of healthcare waste management guidelines, availability of adequate personal protective equipment (PPE), and reporting injury to management were significantly associated with safe infectious healthcare waste management practices (Table 5).

**Table 5:** Factors associated with infectious healthcare waste management practices among waste handlers in Addis Ababa, Ethiopia, 2023 (binary logistic regression analysis, *n* = 379).

Variables	Practice of HIWM		COR at 95% CI	AOR at 95% CI
	Poor	Good		
Sex				
Female	160	212	0.22 (0.026, 1.85)	
Male	1	6	1	
Facility ownership				
Public	149	207	1.52 (0.65, 3.53)	
Private	12	11	1	
Age				
≤ 24	29	55	1.4 (0.75, 2.41)	
25–29	56	60	0.76 (0.45, 1.28)	
30–34	27	34	0.89 (0.48, 1.67)	
> 34	49	69		
Experience				
≤ 2	44	63	0.75 (0.44, 1.26)	1.21 (0.67, 2.19)
3–5	69	63	0.48 (0.29, 0.78)*	0.56 (0.33, 0.97)*
> 6	48	92	1	1
Availability of the guideline				
No	27	17	0.79 (0.40, 1.56)	0.82 (0.39, 1.72)
Yes	40	126	3.95 (2.46, 6.30)*	2.35 (1.21, 4.57)*
I don't know	94	75	1	1
Availability of adequate PPE				
No	117	123	0.49 (0.31, 0.75)*	0.59 (0.35, 0.97)*
Yes	44	95	1	1
Experienced an injury in the last 12 months				
No	135	176	0.81 (0.47, 1.38)	1.19 (0.52, 2.76)
Yes	26	42	1	1
Report your injury to management for mitigation				
No	135	164	0.59 (0.35, 0.98)*	0.50 (0.28, 0.92)*
Yes	26	54	1	1
Availability of newly hired staff				
No	57	135	0.34 (0.22, 0.51)*	1.36 (0.78, 2.35)
Yes	104	83	1	1
The availability of clearly defined procedures				
Yes	57	141	3.34 (2.18, 5.12)*	1.32 (0.69, 2.52)
No	104	77	1	1
Availability of IPC committee				
Yes	103	184	3.05 (1.87, 4.96)*	0.91 (0.38, 2.15)
No	58	34	1	1
Functionality of the IPC committee				
No	60	30	3.72 (2.26, 6.14)*	2.29 (0.98, 5.37)
Yes	101	188	1	1
Knowledge				
Poor	121	143	0.63 (0.40, 0.99)*	0.74 (0.44, 1.23)
Good	40	75	1	1

*Note:* Reference categories are indicated by “1”.

Abbreviations: AOR = adjusted odds ratio, COR = crude odds ratio, CI = confidence interval.

*Statistically significant at *p *< 0.05.

Work experience was significantly associated with HIWM practice. Waste handlers with 3–5 years of experience had lower odds of good HIWM practices compared with those with more than 6 years of experience (AOR = 0.56; 95% CI: 0.33–0.97). However, no statistically significant association was observed among waste handlers with ≤ 2 years of experience compared with those with more than 6 years of experience (AOR = 1.21; 95% CI: 0.67–2.19).

Furthermore, the availability of healthcare waste management guidelines was significantly associated with HIWM practice. Waste handlers working in facilities where guidelines were available had higher odds of good HIWM practices compared with those who were unaware of guideline availability (AOR = 2.35; 95% CI: 1.21–4.57). In contrast, no significant association was observed among waste handlers working in facilities where guidelines were not available compared with those who did not know whether guidelines were available (AOR = 0.82; 95% CI: 0.39–1.72).

Similarly, the availability of adequate PPE was significantly associated with HIWM practice. Waste handlers working in facilities without adequate PPE had lower odds of good HIWM practices compared with those working in facilities where adequate PPE was available (AOR = 0.59; 95% CI: 0.35–0.97).

Additionally, reporting occupational injuries to management was significantly associated with HIWM practice. Waste handlers who did not report occupational injuries to management had lower odds of good HIWM practices compared with those who reported injuries for appropriate mitigation measures (AOR = 0.50; 95% CI: 0.28–0.92) (Table 5).

## 4 Discussion

This study assessed the magnitude of proper infectious healthcare waste management (IWM) practices among waste handlers working in both government and private health facilities in Addis Ababa. The findings revealed that 57.5% (95% CI: 52.4%–63.1%) of participants reported proper IWM practices. Overall, this indicates that nearly half of waste handlers still do not comply with recommended infection prevention and waste management standards, reflecting a substantial occupational and environmental health concern that requires urgent attention. The magnitude observed in this study is comparable with findings reported in other parts of Ethiopia. A study conducted in four public hospitals in Addis Ababa and in South‐Western Ethiopia reported that approximately half of healthcare waste handlers demonstrated good safety practices [8, 18, 20]. Similarly, studies conducted in Gondar and Bahir Dar reported practice levels ranging between 50% and 60% [2, 6]. The similarity may be explained by the shared national healthcare waste management guidelines issued by the Ethiopian Federal Ministry of Health and similar systemic challenges, such as inconsistent supervision, limited resources, and irregular supply of personal protective equipment (PPE). These findings suggest that national‐level policy implementation alone may not be sufficient without strong facility‐level enforcement and continuous monitoring mechanisms. Although the present study included both government and private facilities, the overall regulatory environment and national infection prevention and control (IPC) standards remain the same, which may explain the comparable magnitude. However, the persistence of suboptimal practices across different facility types indicates gaps in the translation of policy into routine practice.

However, the finding of this study is lower than reports from some middle‐income countries. For example, a recent study conducted in India reported relatively high levels of proper healthcare waste management practices among healthcare workers and waste handlers, with magnitudes exceeding 65% [21]. The higher magnitude in such settings may be attributed to stronger regulatory enforcement, structured in‐service training programs, routine monitoring systems, and better institutional accountability mechanisms. This comparison highlights the role of health system strengthening and continuous professional training in improving compliance with waste management standards. In addition, private facilities in some countries are often subject to stricter accreditation requirements, which may enhance compliance. In contrast, although private facilities were included in the present study, variations in enforcement, monitoring, and internal waste management policies across facilities could have influenced the overall moderate result. Furthermore, in the current study, the operational definition of “waste handler” was restricted to cleaners, which may have affected the comparability of findings with other studies that included a broader category of healthcare workers involved in waste handling.

On the other hand, the magnitude observed in this study is higher than findings from some low‐resource settings in sub‐Saharan Africa, where proper healthcare waste management practices among healthcare workers have often been reported below 50%. A systematic review and meta‐analysis across multiple SSA countries estimated an overall prevalence of good practices at 49.7%, with several countries, such as Uganda, Tanzania, and Kenya, reporting levels below 45% [22]. These lower proportions are commonly associated with inadequate infrastructure, lack of proper segregation systems, insufficient training coverage, and limited availability of personal protective equipment (PPE). Compared to rural or resource‐constrained facilities, Addis Ababa, as an urban center, likely benefits from better access to training, improved supervision, and closer regulatory oversight, which may explain the relatively higher magnitude observed in this study. However, despite this relative improvement, the level of unsafe practice continues to be a concern from a public health perspective.

The presence of facility‐level healthcare waste management guidelines was a significant predictor of proper practice in this study. Specifically, waste handlers who were aware of and adhered to these guidelines were more than twice as likely to comply with recommended infectious waste management procedures (AOR = 2.40; 95% CI: 1.23–4.66).

This finding that the availability and use of written guidelines improve compliance with healthcare waste management practices is consistent with evidence from other studies conducted in Ethiopia and sub‐Saharan Africa. These studies similarly report that the presence of structured protocols improves adherence to waste segregation, handling, and disposal procedures among healthcare workers [2, 5, 15, 22, 23].

Adequate personal protective equipment (PPE) availability was strongly associated with proper infectious healthcare waste management in this study (AOR = 0.58; 95% CI: 0.35–0.96), underscoring the critical role of structural resources in promoting safe practices. This finding suggests that behavioral change alone is insufficient without ensuring consistent access to essential protective materials. Consistent access to gloves, masks, boots, and other protective materials enables waste handlers to comply with standard protocols and reduces occupational exposure risks, a finding supported by studies in Addis Ababa and other Ethiopian settings [15, 18, 24].

Work experience also influenced compliance. Waste handlers with longer tenure were more likely to practice proper waste management, possibly due to accumulated knowledge, familiarity with operational procedures, and prior exposure to supervisory feedback. Similar patterns have been observed in Debre Markos, Bahir Dar, and Adama City, where experienced staff demonstrated higher adherence to safe practices compared to less experienced personnel [5, 15, 23, 25]. This highlights the need for continuous on‐the‐job training, especially for newly recruited staff.

Reporting occupational injuries to management was also significantly associated with proper infectious healthcare waste management practice. Waste handlers who did not report occupational injuries to management had lower odds of good HIWM practice than those who reported injuries for appropriate mitigation measures. This finding suggests that timely reporting of occupational injuries may reflect a stronger workplace safety culture, greater awareness of infection prevention practices, and better communication between waste handlers and supervisors. Reporting occupational injuries enables healthcare facilities to investigate incidents, provide appropriate post‐exposure management, identify workplace hazards, and implement corrective measures to prevent similar events in the future [10, 16, 26]. Therefore, encouraging a non‐punitive injury reporting system and ensuring appropriate follow‐up may contribute to improving infectious healthcare waste management practices [16, 26].

Although the availability of an IPC committee, clearly defined waste management procedures, and newly hired staff were associated with safe practice in the bivariable analysis, these associations were no longer statistically significant after adjustment for potential confounders. This suggests that their observed effects in the crude analysis may have been influenced by other related organizational and resource factors rather than representing independent predictors of HIWM practice. For example, the presence of an IPC committee or written waste management procedures alone may not be sufficient to improve practice unless these structures are functional, actively implemented, regularly monitored, and supported by adequate resources [6, 27].

Similarly, the association observed with newly hired staff in the bivariable analysis may reflect differences in facility characteristics, staffing arrangements, or supervision systems rather than the direct effect of staff recruitment status. After adjustment, awareness of healthcare waste management guidelines, availability of adequate PPE, work experience, and reporting of occupational injuries remained important independent predictors. These findings indicate that organizational structures should be accompanied by effective implementation mechanisms, supportive supervision, and availability of necessary resources to achieve sustainable improvements in waste management practices [27].

Knowledge of healthcare waste management was also associated with proper practice in the bivariable analysis; however, this association was attenuated and became statistically non‐significant in the multivariable model. This finding indicates that knowledge alone may not be sufficient to ensure safe infectious waste management practices. Although knowledge provides the foundation for appropriate behavior, waste handlers may experience barriers that limit the application of their knowledge, including inadequate PPE availability, limited access to guidelines, insufficient monitoring, and weak enforcement of procedures. Therefore, improving HIWM practice requires a combination of knowledge‐building interventions and supportive workplace conditions that enable waste handlers to translate knowledge into action [2].

Systemic and operational challenges, including inadequate segregation, irregular waste transport, limited storage infrastructure, and weak enforcement of guidelines, further affect compliance in Addis Ababa and other Ethiopian cities [15, 28, 29]. Knowledge and attitude gaps also play a role; handlers with a better understanding of occupational hazards are more likely to adopt safe practices [2, 24]. These interconnected challenges indicate that infectious healthcare waste management should be addressed using a system‐wide approach rather than isolated interventions.

Collectively, these findings highlight that low compliance is influenced by training, resource availability, guideline enforcement, and experience. From a public health and environmental perspective, improving waste management practices is essential not only for occupational safety but also for reducing broader risks to the community and environment, including contamination and infection transmission. Strengthening institutional policies, ensuring consistent PPE provision, and implementing targeted, role‐specific training programs are critical strategies to improve waste management practices and protect both healthcare workers and the community [20, 23, 30, 31].

## 5 Conclusion

The study indicates that infectious healthcare waste management practices among waste handlers in Addis Ababa remain suboptimal, posing continued risks to occupational safety and public health. Safe infectious healthcare waste management practices were associated with work experience, availability of facility‐level healthcare waste management guidelines, reporting occupational injuries to management, and access to personal protective equipment. Strengthening training programs, ensuring consistent enforcement of guidelines, and improving the regular supply of protective equipment are essential to enhance compliance. Addressing these gaps will contribute to safer healthcare environments and reduce risks to healthcare workers, patients, and the wider community.

### 5.1 Limitations

Despite its strengths, this study has several limitations. First, the cross‐sectional design captures practices and knowledge at a single point in time, limiting causal inferences between associated factors and waste management practices. Therefore, the identified factors should be interpreted as associations and not as causal determinants of infectious healthcare waste management practices. Second, while data on waste handling were collected through direct observation rather than self‐reporting, the short duration of observation may have influenced participants' behavior (Hawthorne effect). Third, the study focused on urban health facilities in Addis Ababa, which may limit generalizability to rural or semi‐urban settings with different resources and operational challenges. Healthcare facilities in rural areas may differ substantially in terms of infrastructure, availability of PPE, staffing levels, supervision, and implementation of healthcare waste management systems. Therefore, the findings may not fully represent waste management practices in other settings, particularly resource‐constrained rural facilities. Future studies including diverse geographic and facility contexts are needed to improve the understanding of healthcare waste management practices across Ethiopia.

## Author Contributions

**Trhas Tadesse Berhe:** writing – review and editing, formal analysis, data curation, validation, methodology, visualization, writing – original draft, investigation, conceptualization, funding acquisition, software, project administration, supervision, resources. **Agezegn Asegid Mekonen:** methodology, validation, visualization, writing – original draft, writing – review and editing, software, formal analysis, data curation. **Mekoya Mengistu Dabulo:** investigation, writing – original draft, writing – review and editing, visualization, methodology, validation, formal analysis, data curation. **Getachew Woldeyohonas Tedila:** investigation, writing – original draft, methodology, validation, visualization, writing – review and editing, software, formal analysis, project administration, data curation, supervision, resources.

## Ethics Statement

The study involving human participants was approved by the Yekatit 12 Hospital Medical College. All procedures were conducted in accordance with local legislation and institutional requirements.

## Consent

Written informed consent was obtained from all participants prior to their inclusion in the study.

## Conflicts of Interest

The authors declare no conflicts of interest.

## Transparency Statement

Trhas Tadesse Berhe affirms that this manuscript is an honest, accurate, and transparent account of the study being reported; that no important aspects of the study have been omitted; and that any discrepancies from the study as planned (and, if relevant, registered) have been explained.

## Data Availability

The datasets generated and/or analyzed during the current study are available from the corresponding author on reasonable request.
